# Supplementary material for: Morphological and Phylogenetic Characterization of Five Novel Nematode-Trapping Fungi (Orbiliomycetes) from Yunnan, China
Source: J Fungi (Basel). 2023 Jul 7;9(7):735. doi: 10.3390/jof9070735 (PMC10381634; doi:10.3390/jof9070735)
Supplement: Supplementary file 1 [file jof-09-00735-s001.zip › jof-2444754-supplementary.pdf]

Table S1. GenBank accession numbers involved in this study. Ex-type strains are in bold. The newly generated sequences are indicated in blue.

Taxon	Strain Number	GenBank accession Number		
		ITS	*TEF*	*RPB2*
Arthrobotrys amerospora	**CBS 268.83**	**NR_159625**	**—**	**—**
Arthrobotrys anomala	YNWS02-5-1	AY773451	AY773393	AY773422
Arthrobotrys arthrobotryoides	CBS 119.54	MH857262	—	—
Arthrobotrys arthrobotryoides	AOAC	MF926580	—	—
Arthrobotrys botryospora	**CBS 321.83**	**NR_159626**	**—**	**—**
Arthrobotrys cladodes	1.03514	MH179793	MH179616	MH179893
Arthrobotrys clavispora	**CBS 545.63**	**MH858353**	—	**—**
Arthrobotrys conoides	670	AY773455	AY773397	AY773426
Arthrobotrys cookedickinson	YMF 1.00024	MF948393	MF948550	MF948474
Arthrobotrys cystosporia	CBS 439.54	MH857384	—	—
Arthrobotrys dendroides	YMF 1.00010	MF948388	MF948545	MF948469
Arthrobotrys dianchiensis	1.00571	MH179720	—	MH179826
Arthrobotrys elegans	1.00027	MH179688	—	MH179797
Arthrobotrys eryuanensis	**CGMCC 3.19715**	**MT612105**	**OM850307**	**OM850301**
Arthrobotrys eudermata	SDT24	AY773465	AY773407	AY773436
Arthrobotrys flagrans	1.01471	MH179741	MH179583	MH179845
Arthrobotrys gampsospora	CBS 127.83	U51960	—	—
Arthrobotrys globospora	1.00537	MH179706	MH179562	MH179814
Arthrobotrys gongshanensis	**CGMCC 3.23753**	**OM801277**	**OM809162**	**OM809163**
Arthrobotrys guizhouensis	YMF 1.00014	MF948390	MF948547	MF948471
Arthrobotrys hengjiangensis	**CGMCC 3.24983**	**OQ946587**	**OQ989312**	**OQ989302**
Arthrobotrys hengjiangensis	**XA190**	**OQ946586**	**OQ989311**	**OQ989301**
Arthrobotrys indica	YMF 1.01845	KT932086	—	—
Arthrobotrys iridis	521	AY773452	AY773394	AY773423
Arthrobotrys janus	85-1	AY773459	AY773401	AY773430

Arthrobotrys javanica	105	EU977514	—	—
Arthrobotrys jindingensis	**CGMCC 3.20895**	**OP236810**	**OP272511**	**OP272515**
Arthrobotrys jinpingensis	**CGMCC 3.20896**	**OM855569**	**OM850311**	**OM850305**
Arthrobotrys koreensis	C45	JF304780	—	—
Arthrobotrys lanpingensis	**CGMCC 3.20998**	**OM855566**	**OM850308**	**OM850302**
Arthrobotrys latispora	H.B. 8952	MK493125	—	—
Arthrobotrys longiphora	1.00538	MH179707	—	MH179815
Arthrobotrys lunzhangensis	**CGMCC 3.20941**	**OK643973**	**OM621809**	**OM621810**
Arthrobotrys luquanensis	**CGMCC 3.20894**	**OM855567**	**OM850309**	**OM850303**
Arthrobotrys mangrovispora	MGDW17	EU573354	—	—
Arthrobotrys megalospora	TWF800	MN013995	—	—
Arthrobotrys microscaphoides	YMF 1.00028	MF948395	MF948552	MF948476
Arthrobotrys multiformis	**CBS 773.84**	**MH861834**	**—**	**—**
Arthrobotrys musiformis	SQ77-1	AY773469	AY773411	AY773440
Arthrobotrys musiformis	1.03481	MH179783	MH179607	MH179883
Arthrobotrys nonseptata	**YMF 1.01852**	**FJ185261**	**—**	**—**
Arthrobotrys obovata	**YMF 1.00011**	**MF948389**	**MF948546**	**MF948470**
Arthrobotrys oligospora	920	AY773462	AY773404	AY773433
Arthrobotrys paucispora	ATCC 96704	EF445991	—	—
Arthrobotrys polycephala	1.01888	MH179760	MH179592	MH179862
Arthrobotrys pseudoclavata	1130	AY773446	AY773388	AY773417
Arthrobotrys psychrophila	1.01412	MH179727	MH179578	MH179832
Arthrobotrys pyriformis	YNWS02-3-1	AY773450	AY773392	AY773421
Arthrobotrys reticulata	CBS 550.63	MH858355	—	—
Arthrobotrys robusta	nefuA4	MZ326655	—	—
Arthrobotrys salina	SF 0459	KP036623	—	—
Arthrobotrys	1.01442	MH179732	MH179580	MH179836

scaphoides				
Arthrobotrys shizishanna	YMF 1.00022	MF948392	MF948549	MF948473
Arthrobotrys shuifuensis	**CGMCC 3.19716**	**MT612334**	**OM850306**	**OM850300**
Arthrobotrys sinensis	105-1	AY773445	AY773387	AY773416
Arthrobotrys sphaeroides	1.01410	MH179726	MH179577	MH179831
Arthrobotrys superba	127	EU977558	—	—
Arthrobotrys thaumasia	917	AY773461	AY773403	AY773432
Arthrobotrys tongdianensis	**CGMCC 3.20942**	**OP236809**	**OP272509**	**OP272513**
Arthrobotrys vermicola	629	AY773454	AY773396	AY773425
Arthrobotrys weixiensis	**CGMCC 3.24984**	**OQ946585**	**OQ989310**	**OQ989300**
Arthrobotrys weixiensis	**FA675**	**OQ946584**	**OQ989309**	**OQ989299**
Arthrobotrys xiangyunensis	**YXY10-1**	**MK537299**	**—**	**—**
Arthrobotrys yunnanensis	**YMF 1.00593**	**AY50993**	**—**	**—**
Arthrobotrys zhaoyangensis	**CGMCC 3.20944**	**OM855568**	**OM850310**	**OM850304**
Dactylellina cangshanensis	**CGMCC 3.19714**	**MK372062**	**MN915115**	**MN915114**
Dactylellina copepodii	**CBS 487.90**	**U51964**	**DQ999835**	**DQ999816**
Dactylellina mammillata	**CBS 229.54**	**AY902794**	**DQ999843**	**DQ999817**
Dactylellina yushanensis	**CGMCC 3.19713**	**MK372061**	**MN915113**	**MN915112**
Drechslerela dactyloides	expo-5	AY773463	AY773405	AY773434
Drechslerella anchonia	CBS 109.37	AY965753	—	—
Drechslerella aphrobrocha	YMF 1.00119	MF948397	—	MF948477
Drechslerella bembicodes	1.01429	MH179731	—	MH179835
Drechslerella brochopaga	CBS 218.61	U51950	—	—
Drechslerella brochopaga	BCRC 34361	FJ380936	—	—
Drechslerella coelobrocha	FWY03-25-1	AY773464	AY773406	AY773435
Drechslerella dactyloides	1.00031	MH179690	MH179554	MH179799
Drechslerella daliensis	**CGMCC 3.20131**	**MT592896**	**OK556701**	**OK638157**

Drechslerella doedycoides	YMF 1.00553	MF948401	—	MF948481
Drechslerella doedycoides	CBS 175.55	MH857432	—	—
Drechslerella effusa	YMF 1.00583	MF948405	MF948557	MF948484
Drechslerella effusa	CBS 774.84	MH861835	—	—
Drechslerella hainanensis	**YMF 1.03993**	**KC952010**	**—**	**—**
Drechslerella heterospora	YMF 1.00550	MF948400	MF948554	MF948480
Drechslerella pengdangensis	**CGMCC 3.24985**	**OQ946589**	**OQ989314**	**OQ989304**
Drechslerella pengdangensis	**DL53**	**OQ946588**	**OQ989313**	**OQ989303**
Drechslerella polybrocha	CCRC 32872	U51973	—	—
Drechslerella polybrocha	DHP 133	U72606	—	—
Drechslerella polybrocha	H.B. 8317	KT222361	—	—
Drechslerella stenobrocha	YNWS02-9-1	AY773460	AY773402	AY773431
Drechslerella tianchiensis	**CGMCC 3.24986**	**OQ946591**	**OQ989316**	**OQ989306**
Drechslerella tianchiensis	**XJ353**	**OQ946590**	**OQ989315**	**OQ989305**
Drechslerella xiaguanensis	**CGMCC 3.20132**	**MT592900**	**OK556699**	**OK638159**
Drechslerella yunlongensis	**CGMCC 3.20946**	**OM956086**	**OQ989318**	**OQ989308**
Drechslerella yunlongensis	**YL402**	**OQ946592**	**OQ989317**	**OQ989307**
Drechslerella yunnanensis	1.01863	MH179759	—	MH179861
Drechslerella yunnanensis	YMF 1.03216	HQ711927	—	—
Orbilia jesu-laurae	**LQ59a**	**MN816816**	**—**	**—**
Orbilia orientalis	H.B.9925	KT222412	—	—
Orbilia orientalis	H.B.9965	KT380104	—	—
Orbilia pseudopolybrocha	**YMF 1.02660**	**NR_172380**	**—**	**—**
Orbilia tonghaiensis	**YMF 1.03006**	**NR_172397**	**MF948570**	**MF948496**
Vermispora fusarina	YXJ02-13-5	AY773447	AY773389	AY773418
Vermispora leguminacea	AS 6.0291	DQ494376	—	—

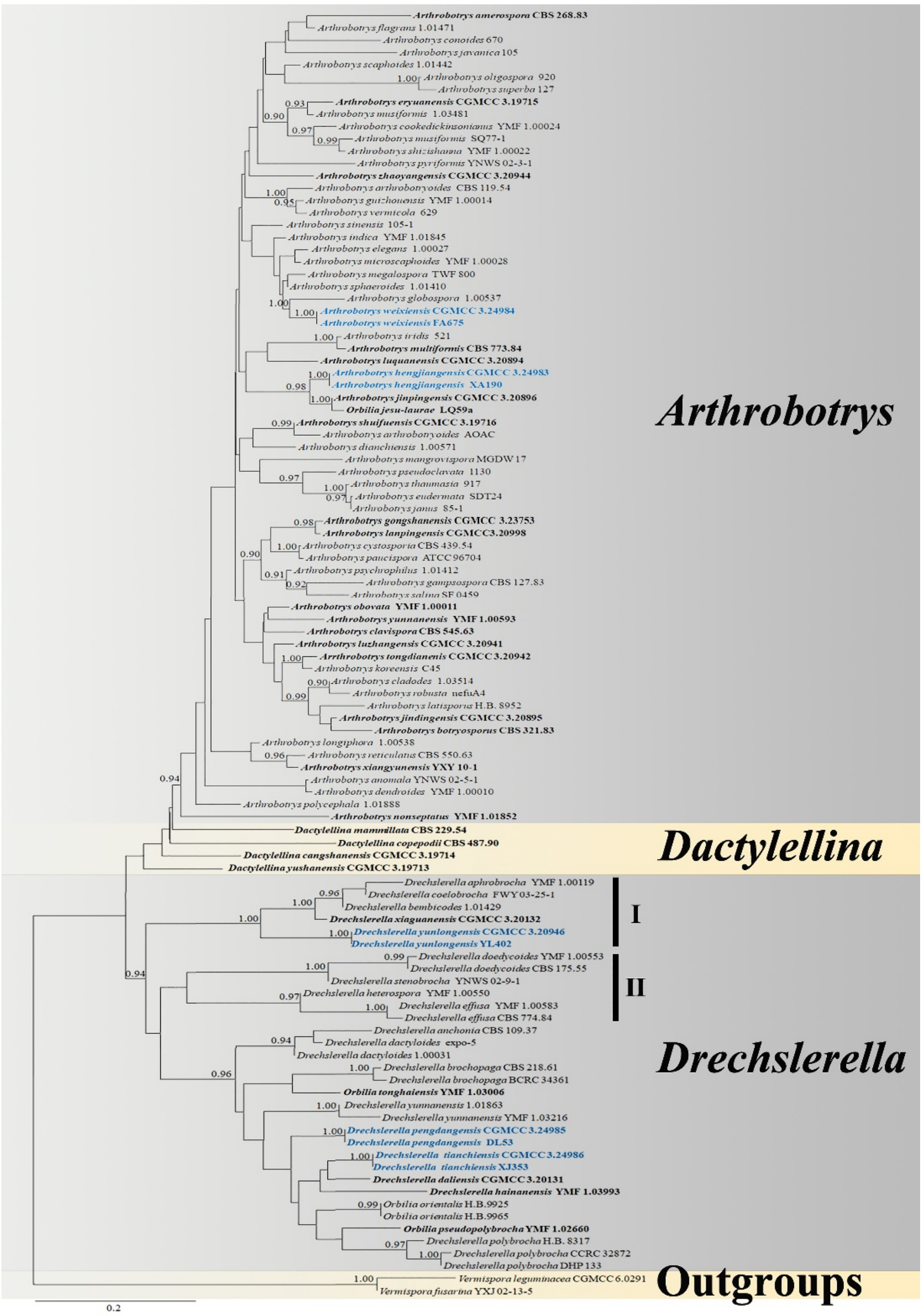

Figure S1. Bayesian majority rule consensus tree based on a combined *ITS*, *TEF* and *RPB2* sequence data from 87 species of Orbiliaceae nematode-trapping fungi. Bayesian posterior probabilities values equal or greater than 0.90 are indicated above the nodes. The new isolates are in blue, type strains are in bold. The tree is rooted by *Vermispora fusarina* YXJ02-13-5 and *V. leguminacea* AS 6.0291.
